# Self-report data as a tool for subtype identification in genetically-defined Parkinson’s Disease

**DOI:** 10.1038/s41598-018-30843-6

**Published:** 2018-08-28

**Authors:** Ashley R. Winslow, Craig L. Hyde, Jemma B. Wilk, Nicholas Eriksson, Paul Cannon, Melissa R. Miller, Warren D. Hirst

**Affiliations:** 1 Human Genetics and Computational Biomedicine, Pfizer Worldwide Research and Development, 1 Portland Street, Cambridge, MA 02139 USA; 2 Early Clinical Development, Pfizer Worldwide Research and Development, 558 Eastern Point Rd., Groton, CT 06340 USA; 3 Neuroscience, Pfizer Worldwide Research and Development, 1 Portland Street, Cambridge, MA 02139 USA; 4 23andMe Inc., 899W. Evelyn Avenue, Mountain View, CA 94041 USA; 5 Human Genetics and Computational Target Validation, Pfizer Worldwide Research and Development, 1 Portland Street, Cambirdge, MA 02139 USA; 6 Present Address: Orphan Disease Center, University of Pennsylvania, 125 S. 31st St., Philadelphia, PA 19104 USA; 7 Present Address: Parkinson’s Disease and Movement Disorders Research, Biogen Research and Early Development, 115 Broadway, Cambridge, MA 02142 USA

## Abstract

Through a targeted recruitment 23andMe has collected DNA and patient-reported symptoms from more than 10,000 subjects reporting a physician-verified diagnosis of PD. This study evaluated the potential of self-report, web-based questionnaires to rapidly assess disease natural history and symptomology in genetically-defined PD populations. While average age-at-diagnosis was significantly lower in *GBA* mutation carriers compared to idiopathic PD, or iPD (idiopathic PD, defined as no *GBA* mutations and no *LRRK2* G2019S mutation), there were no significant differences in symptoms. Conversely, *LRRK2* G2019S carrier status significantly associated with reporting of milder daily symptoms of lightheadedness and several differences were observed at a false discovery rate < 0.1, including increased reporting of changes in walking as an initial symptom of disease, decreased reporting of lightheadedness upon standing, and milder symptoms related to daily functioning. The subclinical differences in symptoms reported by *LRRK2* G2019S carriers suggest differences in underlying pathophysiology and/or disease progression in *LRRK2* carriers compared to iPD. Importantly, we confirm previous findings in PD genetic subsets where disease characteristics were ascertained through clinical exam. Overall, these data support the effective use of self-report and genetic data to rapidly analyze information from a large disease population or difficult to identify genetic subgroups.

## Introduction

While recent GWAS have identified more than 40 loci associated with Parkinson’s disease (PD) susceptibility^1,2^, most of our current biological understanding of the disease stems from research on rare mutations associated with PD. Two such genes, *LRRK2 (leucine rich repeat kinase 2)* and *GBA (*glucosidase beta acid, glucocerebrosidase), are recognized risk loci and known causes of autosomal dominant PD. Identified through genome-wide association studies (GWAS) as well as linkage studies of familial PD, mutations in these genes are not fully penetrant and increase risk of PD in an age-dependent manner^2–8^. The use of *GBA* and *LRRK2* mutations to model pathobiology in research and drug discovery has enabled researchers to gain increased understanding of cellular processes compromised by these mutations. The hope is that these same cellular processes are the basis of disease for all forms of Parkinson’s disease. Despite this progress, the utility of rare, single gene mutations to model complex disorders has been called into question. Previous studies of *GBA* and *LRRK2* mutation carriers suggest that the manifestation of PD in these populations may differ from those with no known genetic cause, or idiopathic PD (iPD). Earlier age-at-onset is reported for both groups, while differences in cognition and depression rates are common in the *GBA* mutation carriers with PD, and gait dysfunction and postural instability, as well as, a more mild disease progression may be more common in the *LRRK2* mutation carriers than that seen in iPD^6,9–12^. The data from these mutaitons suggest distinct differences in the underlying disease pathobiology, calling into question their ability to accurately model more common forms of PD.

*GBA* mutations are implicated in roughly 3–20% (including estimates from general disease population, ethnically-enriched, and familial studies) of iPD populations through a series of coding mutations believed to cause a loss of protein function^7,12–14^. The presence of biallelic *GBA* mutations results in the lysosomal storage disorder Gaucher’s disease, while the heterozygous state significantly increases risk for developing PD. *LRRK2* mutations are far less common, ~1% in the sporadic PD population, and thought to cause disease through a gain-of-function resulting in increased kinase activity^15^. G2019S is the most commonly PD- associated *LRRK2* mutation. Although many pathogenic mutations have been identified in both genes, the mutations have incomplete penetrance as evidenced by healthy mutation carriers and the identification of these two genes as risk factors for PD in studies of idiopathic PD. Incomplete penetrance of *GBA* and *LRRK2* mutations can obscure the heritable nature of these mutations resulting in their contribution to sporadic cases of PD. Gaining a better clinical and genetic understanding of carriers of *LRRK2* and *GBA* mutations will help elucidate similarities in pathogenic events in PD as well as identify disease subtypes that may have distinct biology or distinct clinical manifestation.

Gathering data to adequately power genetic association studies from disease subpopulations or genetically-defined groups can be difficult due to geographical limitations and the cost and accessibility of clinical assessments. In this study we present novel findings from a study of patient-reported symptoms related to Parkinson’s disease in the 23andMe PD research database. We utilize these data to test for association of specific genetic subgroups of PD, namely *GBA* and *LRRK2* pathogenic mutation carriers, with specific disease symptomology, age-at-onset, and differences in medication use. Our findings support the use of patient-reported data and large genetic databases to rapidly test for novel genotype-phenotype associations and are supported by previous studies of clinically-ascertained phenotypes in *GBA* and *LRRK2* carriers. Ultimately, these findings can be used to inform clinical understanding as well as clinical study design, as these endpoints may be more sensitive to the targets of new compounds in development for PD. Also, our results further support the importance and potential power of Patient Centered Outcomes.

## Results

Through a coordinated recruitment, 23andMe (in collaboration with the Michael J. Fox Foundation, the National Parkinson Foundation, and The Parkinson’s Institute and Clinical Center) has recruited more than 10,000 PD participants into their research-focused PD Community cohort, which collects both genetic information and online questionnaire-based assessments. Subjects recruited through this process have reported a physician diagnosis of PD and completed questionnaires that range from normal daily functioning to disease symptoms and severity.

### *GBA* mutation carriers have an earlier age of disease onset

To better understand if carriers of GBA and LRRK2 mutations with PD represent a specific disease subtype, we evaluated differences in disease natural history and manifestation in three genetically-defined groups: *LRRK2* G2019S carriers, carriers of 17 mutations in *GBA* (Table 1), and iPD (defined in this instance as the PD population not carrying any of the before mentioned *GBA* or *LRRK2* variants). From the PD Community, 6,894 individuals completed detailed surveys assessing disease onset and current manifestation and a total of 6,883 were included in our analysis, we did not include individuals carrying both *GBA* and *LRRK2* mutations. We tested for differences in the association of each genetically-defined group with the following conditions: age-at-diagnosis, sex distribution, first symptoms of disease, general symptoms since onset and severity of symptoms, daily-living/functioning (similar to the Unified Parkinson’s Disease Rating Scale (UPDRS) II and I), medications, and the presence of comorbidities. The distribution for sex and current age is shown in Table 2. *GBA* mutation carriers and idiopathic PD groups had a higher percentage of males reporting a diagnosis of PD than females, while *LRRK2* G2019S carriers showed no sex effect, consistent with a recent meta-analysis^16^. While the current age-at-questionnaire distribution is similar between the iPD and *GBA* groups, there are more *GBA* mutation carriers under 60 years old compared to the iPD and *LRRK2* groups, consistent with an earlier age-of-disease-onset. There is no significant difference in the occurrence of type 2 diabetes, ulcerative colitis or Crohn’s disease, or any autoimmune diseases between groups (Supplementary Table 1).

**Table 1:** *LRRK2* and *GBA* mutations used to define *LRRK2* and *GBA* mutation carrier groups, respectively.

rsid or 23andMe SNP code	Mutation (with respect to protein sequence)
***LRRK2***	
rs34637584	G2019S
***GBA***	
rs76763715	N370S
i4000417	84GG
rs1064651	D409H
rs80356771	R463C
rs80356769	V394L
rs80356773	R496H
rs75548401	T369M
rs75671029	D443N
rs74500255	F216Y
rs78396650	A309V
rs364897	N189S
rs80356763	R131L
rs121908311	G377S
rs147138516	D140H
rs2230288	E326K
rs121908295	P454R
rs1141811	T43I

SNPs included in analysis are identified by either rsid or 23andMe code (denoted by “i”, as well as positional naming of the mutation in reference to location in the protein sequence).

**Table 2:** Demographics of the PD Community.

	*LRRK2* G2019S carriers	*GBA* mutation carriers	Idiopathic PD
Total (n = )*	145	262	6476
**Age**, **%**			
under 30	0.0%	0.0%	0.1%
30–45	0.0%	3.8%	3.6%
45–60	20.7%	32.1%	23.3%
60+	79.3%	64.1%	73.0%
**Sex**, **%**			
Male	46.9%	63.0%	60.1%
Female	53.1%	37.0%	39.9%

*Of the 6,894 individuals with a PD diagnosis that completed detailed questionnaires, eleven individuals were dual GBA mutation carriers and LRRK2 G2019S carriers. These individuals were not included in the analysis which ultimately included data from a total of 6,883 individuals.

Compared to the iPD group, average age-at-diagnosis and age-at-symptom-onset are significantly earlier for *GBA* mutation carriers (β = −3.53 (−4.91, −2.91) years, *p* = 5.10 × 10^−7^; and β = −3.58 (−5.09, −2.10) years, *p* = 2.2 × 10^−6^, respectively) but not significant for *LRRK2* mutation carriers (FDR > 0.05 and FDR > 0.1, respectively). Prevalence of PD in G2019S carriers in the 23andMe population was 58.67% for subjects 50–60 years old and 42.86% for those 70–80 years comparing age-at-diagnosis in affected *LRRK2* G2019S to age-at-survey for unaffected carriers in the PD Community and the broader 23andMe research database. The overall prevalence of PD for all *GBA* mutation carriers is lower, with an estimate of 17.84% in the 50–60-year-old age group and 6.98% for those between 70–80 years old. The low prevalence in this group may be affected by the inclusion of less pathogenic *GBA* mutations such as T369M and E326K variants^12,13,17^ or the previously uncited D140H or P454R, and the exclusion of L444P carriers. The L444P mutation cannot be accurately detected via standard SNP array due to homology of the *GBA* pseudogene (*GBAP*).

### *LRRK2* G2019S carriers present with significant differences in disease characteristics

To determine if there are differences in disease symptomology between the genetically-defined disease groups and iPD, we tested the association of each group with a broad spectrum of phenotypes relevant to motor functions and daily living in PD patients.

We tested for differences in self-report of symptom severity related to daily living for symptoms experienced during the week prior to the subject taking the survey. While the *GBA* mutation group showed no significant differences in symptomology compared to the iPD group, *LRRK2* G2019S carriers report significantly milder symptoms of lightheadedness. In addition, several symptoms were reported as milder for *LRRK2* G2019S carriers at FDR < 0.1: excess salivation, fatigue, problems eating, constipation, problems with handwriting, bladder control, and problems dressing (nominally significant) compared to the iPD group at a comparable time after symptom onset (Table 3).

**Table 3:** Comparison of differences in disease manifestation (characteristics) between genetically-defined disease groups and idiopathic disease.

	*LRRK2* G2019S carrier vs iPD			*GBA* mutation carrier vs iPD		
	effect size	p-value	FDR	effect size	p-value	FDR
Diagnosed age, Beta (SE), years	−2.2885 (0.9712)	0.018	0.066	−3.5347 (0.7029)	5.1 × 10^−7^	1.68 × 10^−5^
Symptom age, Beta (SE), years	−1.7425 (1.0445)	0.095	0.174	−3.5841 (0.7559)	2.2 × 10^−6^	3.63 × 10^−5^
**First Symptoms of Disease (Q: What were the first symptoms of PD you experienced? Please check all that apply)**						
Changes in walking, OR (CI)	1.733 [1.217,2.466]	0.0023	0.025	1.008 [0.774,1.312]	0.95	0.980
Changes in handwriting, OR (CI)	0.593 [0.392,0.898]	0.014	0.066	0.934 [0.712,1.225]	0.62	0.880
Tremor or shaking, OR (CI)	0.658 [0.462,0.936]	0.020	0.066	0.985 [0.756,1.283]	0.91	0.980
Soft voice or speech, OR (CI)	0.718 [0.419,1.230]	0.230	0.345	0.88 [0.613,1.263]	0.49	0.880
Balance problems or falling, OR (CI)	1.248 [0.804,1.936]	0.320	0.422	0.743 [0.506,1.092]	0.13	0.578
Other symptoms, OR (CI)	1.038 [0.701,1.538]	0.850	0.935	1.127 [0.855,1.487]	0.4	0.776
Slow movement, OR (CI)	0.982 [0.667,1.447]	0.930	0.959	1.01 [0.766,1.332]	0.94	0.980
**General Symptoms (Q: Have you experienced any of the following symptoms at any time since you developed PD?)**						
Lightheaded, OR (CI)	0.542 [0.344,0.852]	7.9 × 10^−3^	0.045	1.072 [0.816,1.409]	0.62	0.880
Falling, OR (CI)	1.506 [1.054,2.154]	0.025	0.075	1.019 [0.780,1.332]	0.89	0.980
Tremor, OR (CI)	0.676 [0.451,1.014]	0.058	0.120	0.998 [0.716,1.391]	0.99	0.990
Urine control, OR (CI)	0.839 [0.588,1.199]	0.340	0.432	1.21 [0.938,1.560]	0.14	0.578
**Current Symptom Severity (Q: For each of the 20 questions about symptoms below**, **mark the answer that best describes the severity of your symptoms over the past week)**						
Lightheaded, Beta (SE)	−0.3654 (0.095)	1.2 × 10^−4^	0.004	−0.0063 (0.0673)	0.93	0.980
Excess saliva, Beta (SE)	−0.348 (0.1058)	0.001	0.017	0.036 (0.0765)	0.64	0.880
Fatigue, Beta (SE)	−0.283 (0.0978)	0.0038	0.031	−0.0239 (0.0707)	0.74	0.977
Problems eating, Beta (SE)	−0.1918 (0.0725)	0.0082	0.045	−0.0039 (0.0524)	0.94	0.980
Constipation, Beta (SE)	−0.2171 (0.09)	0.016	0.066	−0.0811 (0.0651)	0.21	0.660
Problems with handwriting, Beta (SE)	−0.2259 (0.1047)	0.031	0.078	0.0707 (0.0757)	0.35	0.770
Urine control, Beta (SE)	−0.2251 (0.1045)	0.031	0.078	−0.0635 (0.0752)	0.4	0.776
Problems dressing, Beta (SE)	−0.1627 (0.0764)	0.033	0.078	0.0986 (0.0552)	0.074	0.550
Problems bathing, Beta (SE)	−0.1337 (0.0687)	0.052	0.114	0.0875 (0.0497)	0.078	0.550
Problems getting up, Beta (SE)	−0.1557 (0.0849)	0.067	0.130	0.0576 (0.0614)	0.35	0.770
Problems swallowing, Beta (SE)	−0.0925 (0.0655)	0.160	0.264	−0.077 (0.0474)	0.1	0.550
Speech Problems, Beta (SE)	−0.131 (0.0928)	0.160	0.264	0.0431 (0.0671)	0.52	0.880
Sleep, Beta (SE)	0.131 (0.1097)	0.230	0.345	−0.1113 (0.0793)	0.16	0.587
Balance problems, Beta (SE)	0.0982 (0.0884)	0.270	0.387	0.0123 (0.0639)	0.85	0.980
Daytime sleepiness, Beta (SE)	−0.0842 (0.0793)	0.290	0.399	0.0296 (0.0574)	0.61	0.880
Freezing, Beta (SE)	0.0572 (0.0865)	0.510	0.601	0.1016 (0.0626)	0.1	0.550
Tremor symptoms, Beta (SE)	−0.0543 (0.0828)	0.510	0.601	0.035 (0.0587)	0.55	0.880
Problems turning over, Beta (SE)	−0.0275 (0.076)	0.720	0.819	0.062 (0.055)	0.26	0.660
Pain, Beta (SE)	−0.0128 (0.1018)	0.900	0.958	0.091 (0.0736)	0.22	0.660
Problems with hobbies, Beta (SE)	−0.0038 (0.0979)	0.970	0.970	0.0835 (0.0708)	0.24	0.660

Linear regression model used to test for association of genotype with age-at-diagnosis, age-at-symptom onset, and symptom severity. Logistics regression model used to test for association of genotype with differences in first symptom of disease onset and general symptoms of disease. Effect size, (beta and SE = standard error), p-value, and FDR (false discovery rate) were calculated for each comparison group. Significance is determined across *GBA* and *LRRK2* mutation carrier tests based on achieving p < 7.7 × 10^−4^, the Bonferroni threshold for 66 tests. False Discovery Rates (FDRs) were calculated using the Benjamini Hochberg method^25^ separately for the *LRRK2* and GBA groups, and were assigned to each p-value in each list as the lowest FDR for which the p-value would pass from within its list.

*GBA* mutation carriers also showed no significant differences in first symptoms or current and past symptoms (general symptoms), while *LRRK2* G2019S carrier status showed several significant differences at FDR < 0.1. These include increased likelihood of reporting changes in walking as a first symptom of disease, an increased reporting of falling as a general symptom, and a decreased likelihood to report changes in handwriting, tremor, or shaking as initial symptoms of disease. Full results with applied FDRs are shown in Table 3.

### Medication usage by *GBA* and *LRRK2* mutation carriers

As a potential indicator of disease manifestation or disease course, we tested for association of each disease group with differences in current and past medication use. No findings achieved FDR < 0.05. Of the results nominally significant (p < 0.05) without multiple test adjustment, *LRRK2* mutation carrier status was associated with increased likelihood to report current use of Symmetrel (weak dopamine agonist), Sinemet CR (levodopa carbidopa controlled release), and Sinemet than the iPD population (Supplementary Table 2) and more likely to report past and/or current use of Symmetrel, Parlodel (dopamine agonist), Sinemet CR, Tasmar (COMT inhibitor), Sinemet, and Apokyn injections (dopamine agonist). Interestingly, the *GBA* mutation carrier status was associated with increased likelihood to currently take Sinemet controlled release (levodopa carbidopa), Requip (dopamine agonist), Cogentin (anticholinergic), and Azilect (monoamine oxidase inhibitor), but there were no differences in medication usage when looking at reporting of both past medication and current use. The differences seen in current medications used by *GBA* mutation carriers may be the result of an earlier disease onset and therefore a longer history of disease and medication use.

## Discussion

This study evaluated the potential of web-based questionnaires to rapidly assess disease natural history and symptomology in difficult to access genetically-defined populations. By utilizing data collected from the 23andMe PD community, we were able to rapidly identify and evaluate individuals with specific DNA variants. Findings in these community-derived groups replicated previous studies from clinically-ascertained datasets.

The primary focus of this study was to evaluate the utility of a self-report dataset to identify disease subtypes of genetically-defined populations. While *GBA* mutation carriers had an earlier age-at-diagnosis on average, only the *LRRK2* G2019S carriers presented with significant differences in motor phenotypes and daily functioning, *GBA* mutation carriers were indistinguishable from the iPD group. Overall, our findings suggest *LRRK2* G2019S carriers represent a subtype of PD characterized by more mild general disability in daily living activities or a slower disease progression, with more changes in walking as an initial disease symptom than the general PD population. Similar findings have been reported in studies of *LRRK2* G2019S carriers with Ashkenazi Jewish ancestry^18^, in early-onset PD^11^, and in iPD cohorts^19^, as well as reports of gait disturbances or gait variability in asymptomatic *LRRK2* G2019S carriers^20^. Previous findings suggest differences seen in *LRRK2* mutation carriers are a result of early-onset disease or are due to enrichment of unique Ashkenazi-specific genetics rather than pathogenic events specific to *LRRK2*. Studies done in a variety of different mutation carrier groups have controlled for these differences (early-onset disease, Ashkenazi Jewish PD, and even non-PD mutation carriers) providing further evidence that these differences are central to *LRRK2* dysregulation^11,18–20^. These studies examined similar functional motor endpoints but were measured in a clinical setting and therefore are likely to be more accurate and sensitive than subjective responses collected by patient reporting. Importantly, the ability of this study to replicate these previous findings encourages the continued use and development of carefully designed patient-based questionnaires to quickly access large or genetically specified groups of subjects and demonstrates the power of online surveys in a large genotyped cohort. We also discovered a novel association of *LRRK2* mutation carrier status with milder symptoms associated with lightheadedness compared to the sporadic PD population. An analysis of medication use did not produce results passing a strict threshold for multiple test correction (FDR < 0.05) but there were nominally significant results (pvalue < 0.05) that warrant future study. Importantly, future studies should expand data collection to include medication dosage as well as interventional treatment with deep brain stimulation.

A potential weakness of our study is the aggregation of *GBA* mutations into one group, compared to the single variant analysis conducted for *LRRK2*. The aggregation for *GBA* was necessary due to the relatively small population size for each individual mutation. This was in contrast to the G2019S *LRRK2* mutation, which was sufficiently powered (n = 145 individuals with PD). While the assessment of mutations as a single group (*GBA* mutation group) rather than as individual mutations may introduce additional heterogeneity, we expect that if all mutations assessed are pathogenic they will likely contribute to the PD phenotype through similar molecular mechanisms (i.e. loss of function or common effect on key protein-protein interactions or pathways). Our findings are also in agreement with previous reports showing no significant difference in disease symptoms in *GBA* mutation carriers, except for early occurrence of dementia in this population^10,21^, which we did not test in our analysis. Future characterization of residual enzymatic activity associated with each mutation will allow for more refined correlation analyses between disease-relevant endpoints and enzyme activity. These analyses would further test the hypothesis that loss of glucocerebrosidase is the causative molecular event resulting from GBA pathogenic mutations in PD.

Overall, we identified phenotypic differences in the *LRRK2* carriers that may reflect differences in the underlying pathophysiology of disease, suggesting that genetically-defined disease populations may be one method for identifying disease subtypes or endophenotypes within the broader spectrum of disease.

It is important for researchers to consider potential biases of human-based research in the application of these results to future research or study design. While biases were not studied in this dataset directly, research participants in human-based studies are often Caucasian, more affluent, and attend school longer than those in the general population. This can ultimately result in findings that are not applicable in a real-world setting. The participants in this study were recruited through a variety of channels including the 23andMe research database as well as a targeted email campaign in collaboration with the Michael J. Fox Foundation, The Parkinson’s Institute and Clinical Center, and numerous other PD patient groups and clinics. Given the facility of web-based surveys, future studies should strive to diversify participation and seek to conduct research that reflects the heterogeneity of the general population.

These differences have important implications for clinical study design. As the first phase of *LRRK2* kinase inhibitors may soon be tested in early trials of safety and efficacy, a clear understanding of the patient population will provide better opportunities for success by assessing differences in the target population’s unique clinical manifestation and disease decline trajectory. It is logical to hypothesize that the clinical phenotypes most closely linked to the therapeutic target (i.e. phenotypes identified as early symptom presentations in genetically-defined population where that gene is targeted by investigational drugs) are the phenotypes that are most likely to respond to the therapy with early intervention. Importantly, the identification of disease subtypes does not imply that therapeutic success in this population for this target is limited to the subpopulation. The advantage of subpopulation studies is to provide an early indicator of efficacy in a more homogenous population. From this point, early biomarker studies or sub-group analyses could enable careful expansion of the therapy, from use in a genetically-defined population into non-mutation carrier groups.

Overall our data support the use of clinically-relevant patient questionnaires to quickly access genetically predefined subpopulations and evaluate disease relevant differences. Ideally, future clinical trials will test therapeutic efficacy of new treatments in prodromal PD populations and the ability of treatments to delay or prevent the onset of significant motor symptoms. As these populations grow large enough for sufficiently powered studies, evaluation of genetic markers and phenotypic traits paired with collection of biomarker data will enable early identification of prodromal patient populations for prevention studies.

## Methods

### Study Design and Population (23andMe PD Community)

Research participants were recruited by 23andMe, Inc., a direct-to-consumer genetics company. The 23andMe PD cohort was described in detail previously^22^. Briefly, participants with PD were recruited from a variety of channels, including from the existing 23andMe customer base and through external channels and media, such as a targeted email campaign in conjunction with the Michael J. Fox Foundation, the Parkinson’s Institute and Clinical Center, and numerous other PD patient groups and clinics. All research participants included in the analyses provided informed consent and answered surveys online according to the 23andMe human subjects protocol, which was reviewed and approved by Ethical & Independent Review Services, an AAHRPP-accredited, external institutional review board. Details of questionnaires, which included questions from parts I and II of the MDS UPDRS, are included in Supplemental Information.

### Genotype- phenotype association analyses

We tested if genotype (carrier status of either *LRRK2* G2019S or *GBA* mutations) associated with differences in age-at-diagnosis and age-at-symptom-onset by fitting a linear regression model and adjusting for sex and the first five principal components for genetic ancestry (see ancestry determination methods in^23,24^). The same adjustments were included for association of genotype with symptom severity (scale of 1–5) with the additional adjustment for age and age-at-symptom-onset, also using linear regression. Significance is determined across *GBA* and *LRRK2* mutation carrier tests based on achieving p < 7.7 × 10^−4^, the Bonferroni threshold for 66 tests. False Discovery Rates (FDRs) were calculated using the Benjamini Hochberg method^25^ separately for the *LRRK2* and GBA groups, and were assigned to each p-value in each list as the lowest FDR for which the p-value would pass from within its list. Sets of results with FDR < 0.1 are cited. Symptom questionnaires are described in detail in the Supplemental Information.

We also tested for association of genotype (carrier status of either *LRRK2* G2019S or *GBA* mutations) with binary traits, including differences in current and former medications, first symptoms, general symptoms of disease, and comorbidities using logistic regression and adjusting for age, sex, and the first five principal components for genetic ancestry.

In Table 3, continuous traits assessed by linear regression are given effect sizes in the units of that trait, along with standard error of that effect size (e.g. Beta (SE) as denoted in the table). Binary traits assessed by logistic regression show effect sizes of Odds Ratio per allele, with an associated 95% confidence interval (denoted OR (CI) in the table).

Detailed questionnaires are included in the Supplemental Information.

### Comorbidities

Three phenotypes were tested for association with the occurrence of comorbidities in three categories: any autoimmune disease, type 2 diabetes, and irritable bowel disease (including Crohn’s disease and ulcerative colitis).

### Genotyping, Imputation, and Quality Control

Genotyping, imputation and quality control methods previously described^26^. DNA extraction and genotyping were performed on saliva samples by National Genetics Institute (NGI), a CLIA licensed clinical laboratory and subsidiary of Laboratory Corporation of America. Samples were genotyped on one of four genotyping platforms. The V1 and V2 platforms were variants of the Illumina HumanHap550 + BeadChip, including ~25,000 custom SNPs selected by 23andMe, with a total of ~560,000 SNPs. The V3 platform was based on the Illumina OmniExpress + BeadChip, with custom content to improve overlap with the V2 array, with a total of ~950,000 SNPs. The V4 platform (currently used) is a fully custom array, including a lower redundancy subset of V2 and V3 SNPs with additional coverage of lower-frequency coding variation, and ~570,000 SNPs. Samples that failed to reach 98.5% call rate were re-analyzed. Individuals whose analyses failed repeatedly were re-contacted by 23andMe customer service to provide additional samples, as is done for all 23andMe customers.

## Electronic supplementary material

Supplemental Tables

Supplemental Methods
