# Supplementary material for: Self-report data as a tool for subtype identification in genetically-defined Parkinson’s Disease
Source: Sci Rep. 2018 Aug 28;8:12992. doi: 10.1038/s41598-018-30843-6 (PMC6113219; doi:10.1038/s41598-018-30843-6)
Supplement: Supplementary file 1 — Supplemental Tables [file 41598_2018_30843_MOESM1_ESM.doc]

**Self-report data as a tool for subtype identification in genetically-defined Parkinson’s Disease**

**Winslow, A.R.** ^1, 4^**, Hyde, C.L.** ^2^**, Wilk, J.B.** ^1,4^**, Eriksson, N.** ^5,6^**, Canon, P.** ^5^**, Miller, M.R.** ^1^**, Hirst, W. D.** ^3,4^

1. Pfizer Global Research and Development, Human Genetics and Computational Biomedicine, 610 Main Street, Cambridge, MA 02139
2. Pfizer Global Research and Development, Statistics, 610 Main Street, Cambridge, MA 02139
3. Pfizer Global Research and Development, Neuroscience, 610 Main Street, Cambridge, MA 02139
4. Previous Pfizer affiliation
5. 23andMe Inc., 899 W. Evelyn Avenue, Mountain View, California 94041, USA
6. Previous 23andMe affiliation

**SUPPLEMENTARY TABLES**

**Supplementary Tables 1-2**

**Supplementary Tables**

**Supplementary Table 1.** Genotype association with differences in comorbidities by logistic regression.

|  | ***LRRK2* G2019S carrier, OR [CI]** | **pval (*LRRK*2 vs iPD)** | ***GBA* mutation carriers, OR [CI]** | **pval (*GBA* vs iPD)** |
| --- | --- | --- | --- | --- |
| Type 2 Diabetes | 1.468 [0.736,2.927] | 0.28 | 0.728 [0.364,1.456] | 0.37 |
| Crohn's Disease or Ulcerative Colitis | 1.723 [0.616,4.817] | 0.3 | 0.467 [0.112,1.953] | 0.3 |
| Any Autoimmune | 1.055 [0.642,1.734] | 0.83 | 0.663 [0.426,1.031] | 0.068 |
| Effect size is expressed as odds ratio per allele (OR) with an associated 95% confidence interval (CI). | | | | |

**Supplementary Table 2.** Genotype association with differences in past PD medication use by logistic regression.

|  | ***LRRK2* G2019S carrier vs iPD** | | | ***GBA* mutation carriers vs iPD** | | |
| --- | --- | --- | --- | --- | --- | --- |
|  | **OR [CI]** | **pvalue** | **FDR** | **OR [CI]** | **pvalue** | **FDR** |
| **Current Medications** | | | | | | |
| symmetrel | 2.086 [1.204,3.616] | 0.0088 | 0.12 | 1.166 [0.737,1.843] | 0.51 | 0.92 |
| sinemet cr | 1.689 [1.061,2.689] | 0.027 | 0.18 | 1.447 [1.024,2.043] | 0.036 | 0.36 |
| sinemet | 1.558 [1.040,2.334] | 0.031 | 0.18 | 0.969 [0.724,1.296] | 0.83 | 0.99 |
| comtan | 1.929 [0.946,3.936] | 0.071 | 0.21 | 0.862 [0.446,1.666] | 0.66 | 0.99 |
| requip | 1.498 [0.963,2.333] | 0.073 | 0.21 | 1.505 [1.097,2.064] | 0.011 | 0.36 |
| stalevo | 1.465 [0.858,2.502] | 0.16 | 0.43 | 1.172 [0.774,1.774] | 0.45 | 0.90 |
| apokyn injection | 2.976 [0.541,16.379] | 0.21 | 0.46 | 1.039 [0.129,8.355] | 0.97 | 0.99 |
| cogentin | 3.965 [0.432,36.434] | 0.22 | 0.46 | 3.927 [1.097,14.053] | 0.035 | 0.36 |
| parcopa | 1.574 [0.676,3.663] | 0.29 | 0.53 | 1.33 [0.651,2.718] | 0.43 | 0.90 |
| selegiline | 1.282 [0.558,2.942] | 0.56 | 0.93 | 0.787 [0.377,1.643] | 0.52 | 0.92 |
| razadyne | 1.593 [0.177,14.369] | 0.68 | 0.97 | 0 [0.000, Inf] | 0.99 | 0.99 |
| tasmar | 1.432 [0.168,12.238] | 0.74 | 0.99 | 2.123 [0.607,7.416] | 0.24 | 0.64 |
| mirapex | 0.958 [0.599,1.532] | 0.86 | 1.00 | 1.028 [0.733,1.442] | 0.87 | 0.99 |
| azilect | 0.974 [0.642,1.476] | 0.9 | 1.00 | 1.404 [1.047,1.883] | 0.023 | 0.36 |
| neupro | 1.081 [0.131,8.937] | 0.94 | 1.00 | 1.26 [0.286,5.548] | 0.76 | 0.99 |
| exelon | 1.043 [0.236,4.611] | 0.96 | 1.00 | 1.991 [0.818,4.843] | 0.13 | 0.64 |
| aricept | 0 [0.000, Inf] | 0.97 | 1.00 | 1.649 [0.816,3.331] | 0.16 | 0.64 |
| artane | 0 [0.000, Inf] | 0.97 | 1.00 | 1.058 [0.418,2.682] | 0.9 | 0.99 |
| parlodel | 0 [0.000, Inf] | 0.99 | 1.00 | 0 [0.000, Inf] | 0.99 | 0.99 |
| permax | 0 [0.000, Inf] | 1 | 1.00 | 0 [0.000, Inf] | 0.99 | 0.99 |
| **Current and Past Medications** | | | | | | |
| symmetrel | 1.887 [1.187,3.001] | 0.0072 | 0.12 | 1.244 [0.863,1.792] | 0.24 | 0.64 |
| parlodel | 3.49 [1.376,8.855] | 0.0085 | 0.12 | 1.197 [0.417,3.434] | 0.74 | 0.99 |
| sinemet cr | 1.602 [1.058,2.427] | 0.026 | 0.18 | 1.223 [0.894,1.672] | 0.21 | 0.64 |
| tasmar | 2.621 [1.085,6.328] | 0.032 | 0.18 | 1.186 [0.502,2.803] | 0.7 | 0.99 |
| sinemet | 1.563 [1.022,2.392] | 0.039 | 0.18 | 1.013 [0.756,1.357] | 0.93 | 0.99 |
| apokyn injection | 2.744 [1.040,7.239] | 0.041 | 0.18 | 1.588 [0.658,3.835] | 0.3 | 0.71 |
| aricept | 0.147 [0.020,1.087] | 0.06 | 0.21 | 1.627 [0.892,2.968] | 0.11 | 0.63 |
| selegiline | 1.574 [0.969,2.557] | 0.067 | 0.21 | 1.015 [0.675,1.525] | 0.94 | 0.99 |
| permax | 1.954 [0.945,4.041] | 0.071 | 0.21 | 1.398 [0.767,2.548] | 0.27 | 0.68 |
| razadyne | 2.909 [0.584,14.502] | 0.19 | 0.46 | 1.616 [0.365,7.166] | 0.53 | 0.92 |
| comtan | 1.383 [0.843,2.270] | 0.2 | 0.46 | 0.922 [0.615,1.383] | 0.7 | 0.99 |
| stalevo | 1.313 [0.824,2.092] | 0.25 | 0.50 | 1.275 [0.908,1.788] | 0.16 | 0.64 |
| parcopa | 1.452 [0.754,2.793] | 0.26 | 0.50 | 1.171 [0.681,2.012] | 0.57 | 0.95 |
| azilect | 0.864 [0.578,1.292] | 0.48 | 0.83 | 1.289 [0.963,1.726] | 0.088 | 0.59 |
| neupro | 1.18 [0.619,2.250] | 0.62 | 0.97 | 0.944 [0.575,1.548] | 0.82 | 0.99 |
| artane | 1.194 [0.572,2.490] | 0.64 | 0.97 | 1.351 [0.827,2.207] | 0.23 | 0.64 |
| mirapex | 1.095 [0.734,1.634] | 0.66 | 0.97 | 0.878 [0.654,1.178] | 0.38 | 0.84 |
| cogentin | 1.235 [0.357,4.269] | 0.74 | 0.99 | 1.09 [0.428,2.776] | 0.86 | 0.99 |
| requip | 1.047 [0.698,1.569] | 0.82 | 1.00 | 1.203 [0.900,1.608] | 0.21 | 0.64 |
| exelon | 1.057 [0.363,3.072] | 0.92 | 1.00 | 1.91 [0.982,3.716] | 0.057 | 0.46 |
| Effect sizes are expressed as odds ratio per allele (OR), with an associated 95% confidence interval (CI). p-value, and FDR (false discovery rate) were calculated for each comparison group. False Discovery Rates (FDRs) were calculated using the Benjamini Hochberg method (Benjamini and Hochberg, 1995) separately for the *LRRK2* and GBA groups, and were assigned to each p-value in each list as the lowest FDR for which the p-value would pass from within its list. | | | | | | |
