# Supplementary material for: Self-report data as a tool for subtype identification in genetically-defined Parkinson’s Disease
Source: Sci Rep. 2018 Aug 28;8:12992. doi: 10.1038/s41598-018-30843-6 (PMC6113219; doi:10.1038/s41598-018-30843-6)
Supplement: Supplementary file 2 — Supplemental Methods [file 41598_2018_30843_MOESM2_ESM.docx]

**Self-report data as a tool for subtype identification in genetically-defined Parkinson’s Disease**

**Winslow, A.R.** ^1, 4^**, Hyde, C.L.** ^2^**, Wilk, J.B.** ^1,4^**, Eriksson, N.** ^5,6^**, Canon, P.** ^5^**, Miller, M.R.** ^1^**, Hirst, W. D.** ^3,4^

1. Pfizer Global Research and Development, Human Genetics and Computational Biomedicine, 610 Main Street, Cambridge, MA 02139
2. Pfizer Global Research and Development, Statistics, 610 Main Street, Cambridge, MA 02139
3. Pfizer Global Research and Development, Neuroscience, 610 Main Street, Cambridge, MA 02139
4. Previous Pfizer affiliation
5. 23andMe Inc., 899 W. Evelyn Avenue, Mountain View, California 94041, USA
6. Previous 23andMe affiliation

**SUPPLEMENTARY INFORMATION**

**SUPPLEMENTARY INFORMATION**

**METHODS**

**Questionnaires**

- Age-at-symptom-onset and age-at-diagnosis questions:

*1) How old were you when you first experienced symptoms of Parkinson’s disease?*

*2) How old were you when you were first diagnosed with Parkinson’s disease?*

- Symptom severity was assessed for each of 20 different symptoms. Participants answered a question asking them to rank the severity of their symptoms (scale 1-5: normal, slight, mild, moderate, severe) over the past week.
- First symptoms of disease

*What were the first symptoms of Parkinson’s disease you experienced? Please check all that apply.*

- Tremor or shaking
- Changes in handwriting
- Soft voice or speech
- Changes in walking
- Slowness of movement
- Balance problems or falling
- Other symptoms
- General symptoms of disease

*Have you experienced any of the following symptoms at any time since you developed Parkinson’s disease? Please check all that apply.*

- Falling
- Trouble with urine control
- Frequently feeling light-headed on standing
- Shaking or tremor
- None of the above
- Medication use questions

*1) The questions in this section will ask about the medications you are taking and have taken in the past. Which of the following Parkinson’s disease medications* have you ever taken*? Please check all that apply.*

*2) Which of the following Parkinson’s disease medications are you* currently *taking? Please check all that apply.*

- - Aricept (donepezil)
  - Artane (trihexyphenidyl)
  - Apokyn injection (apomorphine)
  - Azilect (rasagiline)
  - Cogentin (benztropine)
  - Comtan (entacapone)
  - Eldepryl, Carbex, Atapryl, or Emsam patch (selegiline or deprenyl)
  - Exelon patch (rivastigimine transdermal system)
  - Mirapex (pramipexole)
  - Neupro patch (rotigotine patch)
  - Parcopa (carbidopa/levodopa orally disintegrating tablet)
  - Parlodel (bromocriptine)
  - Permax (pergolide)
  - Razadyne (galantamine)
  - Requip (ropinerole)
  - Sinemet or Atamet (carbidopa/levodopa)
  - Sinemet CR (controlled release carbidopa/levodopa)
  - Stalevo (carbidopa, levodopa and entacapone)
  - Symmetrel (amantadine)
  - Tasmar (tolcapone)
  - Some other Parkinson’s disease medication
  - None of the above
